# Effects of Intertidal Position on Metabolism and Behavior in the Acorn Barnacle, *Balanus glandula*

**DOI:** 10.1093/iob/obab010

**Published:** 2021-04-30

**Authors:** Kali M Horn, Michelle E H Fournet, Kaitlin A Liautaud, Lynsey N Morton, Allie M Cyr, Alyse L Handley, Megan M Dotterweich, Kyra N Anderson, Mackenzie L Zippay, Kristin M Hardy

**Affiliations:** 1 Department of Biological Sciences, Center for Coastal Marine Sciences, California Polytechnic State University, San Luis Obispo, CA 93407, USA; 2 Cornell Lab of Ornithology, Cornell University, Ithaca, NY 14850, USA; 3 Department of Biology, Sonoma State University, Rohnert Park, CA 94928, USA

## Abstract

The intertidal zone is characterized by persistent, tidally-driven fluctuations in both abiotic (e.g., temperature, oxygen, and salinity) and biotic (e.g., food availability and predation) factors, which make this a physiologically challenging habitat for resident organisms. The relative magnitude and degree of variability of environmental stress differ between intertidal zones, with the most extreme physiological stress often being experienced by organisms in the high intertidal. Given that so many of the constantly shifting parameters in this habitat are primary drivers of metabolic rate (e.g., temperature, [O_2_], and food availability), we hypothesized that sessile conspecifics residing in different tidal zones would exhibit distinct “metabolic phenotypes,” a term we use to collectively describe the organisms’ baseline metabolic performance and capacity. To investigate this hypothesis, we collected acorn barnacles (*Balanus glandula*) from low, mid, and high intertidal positions in San Luis Obispo Bay, CA, and measured a suite of biochemical (whole-animal citrate synthase (CS) and lactate dehydrogenase (LDH) activity, and aerial [D-lactate]), physiological (O_2_ consumption rates), morphological (body size), and behavioral (e.g., cirri beat frequency and percentage of time operculum open) indices of metabolism. We found tidal zone-dependent differences in *B. glandula* metabolism that primarily related to anaerobic capacity, cirral activity patterns, and body size. Barnacles from the low intertidal tended to have a greater capacity for anaerobic metabolism (i.e., increased LDH activity and increased baseline [D-lactate]), have reduced cirral beating activity—and presumably reduced feeding—when submerged, and be smaller in size compared to conspecifics in the high intertidal. We did not, however, see any D-lactate accumulation in barnacles from any tidal height throughout 96 h of air exposure. This trend indicates that the enhanced capacity of low intertidal barnacles for anaerobic metabolism may have evolved to support metabolism during more prolonged episodes of emersion or during events other than emersion (e.g., coastal hypoxia and predation). There were also no significant differences in CS activity or baseline O_2_ consumption rates (in air or seawater at 14°C) across tidal heights, which imply that aerobic metabolic capacity may not be as sensitive to tidal position as anaerobic processes. Understanding how individuals occupying different shore heights differ in their metabolic capacity becomes increasingly interesting in the context of global climate change, given that the intertidal zone is predicted to experience even greater extremes in abiotic stress.

## Introduction

Throughout the course of a single tidal cycle, intertidal organisms can experience extreme variation in abiotic and biotic factors. Chronic variability in temperature, oxygen, salinity, pH, food availability, and predation—among other things—can have profound impacts on physiology, survivability, and distribution (Connell 1972; Foster 1986; Solan and Whiteley 2016). While submerged in seawater (immersion), resident organisms experience relatively stable ocean temperatures, O_2_ levels, and access to food. Whereas during periodic air exposure (emersion), they face greater temperature extremes (Helmuth and Hofmann 2001), variable O_2_ availability (Castro et al. 2001), osmotic challenge (Burnett and McMahon 1987), desiccation stress (Foster 1971), and reduced food availability (Gillmor 1982). Thus, tolerance to emersion is critical in shaping the physiology, and ultimately the ecological distribution patterns, of intertidal species (Somero 2002; Tomanek and Helmuth 2002).

Of the abiotic stressors that fluctuate across the tidal cycle, temperature and O_2_ both substantially impact metabolic rate in ectotherms (McMahon 1988; Somero 2002; Pörtner 2010). The relative magnitude and degree of variability of these two environmental stressors, however, can differ greatly between intertidal zones with the most extreme and variable physiological stress often experienced by high intertidal organisms that spend a greater proportion of their time in the air (Connell 1972; Shick et al. 1988; Somero 2002). In a mussel bed during low tide, for example, there can be a roughly 7–8°C difference in average maximum body temperature between mussels anchored in the low and high intertidal zones, and mussels in the high intertidal consistently experience much greater inter-individual variation in body temperature (as much as 14°C between mussels in close proximity; Miller and Dowd 2017). A difference in temperature of this magnitude could easily result in significant differences in baseline O_2_ consumption rates (Newell 1969, 1973) or growth rates (Connor and Robles 2015) for an organism. Prolonged exposure to high temperatures can also lead to decreased survival, although high shore species across many invertebrate groups (e.g., snails, porcelain crabs, and abalone) have been shown to have a greater tolerance to thermal stress than those from lower tidal heights (Tomanek and Somero 1999; Somero 2002, 2010; Stillman 2003).

Oxygen availability across the intertidal varies more unpredictably than temperature. This is because O_2_ levels are influenced by both abiotic variation between air and water (e.g., differences in O_2_ concentration at the same O_2_ partial pressure [pO_2_], or differences in O_2_ between air and water during a coastal hypoxia episode), and biological variation in aerial gas exchange ability between organisms. Intertidal invertebrates differ substantially in their ability to facilitate O_2_ uptake or carbon dioxide loss across their respiratory surfaces while in the air. Some intertidal organisms have a very high capacity for aerial gas exchange owing to substantial cutaneous O_2_ uptake (e.g., stalked barnacles (Petersen et al. 1974) and porcelain crabs (Stillman and Somero 1996)) or O_2_ uptake that occurs within an internal seawater or air filled cavity that houses their respiratory surfaces (e.g., branchial cavity of intertidal crabs (Greenaway et al. 1996) and mantle cavity of acorn barnacles (Anderson 1994)). In these species, aerobic metabolism can continue without interruption during emersion (Newell 1973; Petersen et al. 1974). Other aquatic species, like mussels, implement virtually no gas exchange while out of water due to gill filament clumping and shell/operculum closure for the purposes of desiccation and predation avoidance (Coleman 1973; Newell 1973). The combined effects of increased temperature, which inherently elevates metabolic rates in ectotherms (Huey and Stevenson 1979; Prosser 1991), and decreased O_2_ due to shell closure make air emersion especially challenging for intertidal organisms that do not facilitate aerial O_2_ uptake.

Organisms experience longer periods of emersion with increasing shore height (Finke et al. 2007), and this can lead to differences in morphological phenotypes between species or conspecifics that inhabit distinct intertidal zones. Compared to low intertidal barnacles of the same species, for example, certain high intertidal barnacles have been found to have lighter coloration to prevent heat stress, smaller aperture openings to reduce desiccation stress (Achituv and Borut 1975), and longer cirri to increase food capture during shorter bouts of submersion (Chan and Hung 2005). Other organisms, like mussels (Marsden and Weatherhead 1999), chitons (Otaíza and Santelices 1985), and snails (Brown and Quinn 1988), often have longer or larger shells in lower intertidal positions due to increased food availability.

There is also a small body of evidence for variation in metabolic physiology between species that inhabit discrete vertical zones (e.g., porcelain crabs (Stillman and Somero 1996; Gunderson et al. 2019) and acorn barnacles (Augenfeld 1967; Vial et al. 1999)). Stillman and Somero (1996) observed significant differences in resting metabolic rates, cardiac parameters, and aerial lactate production between closely related species of porcelain crabs characteristic of different tidal heights. Whether such metabolic variation exists within a single population of conspecifics that span the intertidal has received very little attention (Dowd et al. 2013). Since metabolic processes are strongly influenced by temperature and O_2_ availability, and these parameters can vary greatly over the span of less than a meter in the rocky intertidal (Helmuth and Hofmann 2001), metabolic variation due to phenotypic plasticity or selection is likely. We hypothesize that there will predictable differences in metabolic parameters within a single species across their vertical distribution in the intertidal.

Barnacles represent ideal model organisms for assessing how intertidal position influences the basic metabolic strategies and capacities of an organism, a characteristic we collectively refer to as the “metabolic phenotype.” This suitability is due to their ubiquitous presence in the rocky intertidal, their expansive vertical distribution across the subtidal and intertidal zones, as well as their sessile nature. In this project, we aimed to describe the baseline metabolic phenotype of the common acorn barnacle, *Balanus glandula* Darwin 1854, and determine whether there are distinct phenotypes in *B. glandula* anchored at different tidal heights across their broad vertical distribution (Glynn 1965) in the intertidal zone. We chose to measure a suite of parameters that intentionally span several levels of organization (e.g., enzymes to behavior) in an effort to thoroughly characterize metabolic phenotypes in this species. Toward this goal, we collected *B. glandula* from low, mid, and high intertidal zones in San Luis Obispo Bay, CA, and compared biochemical (whole-animal citrate synthase [CS] and lactate dehydrogenase [LDH] activity, aerial [D-lactate]), physiological (O_2_ consumption rates), morphological (body size), and behavioral (e.g., cirri beat frequency [CBF] and percentage of time operculum open) indices of their metabolism. The identification of distinct metabolic phenotypes across the intertidal zone becomes increasingly important in the context of global climate change, given that the intertidal is predicted to experience even greater extremes in temperature, changes in ocean chemistry, and more frequent hypoxic events (Harley et al. 2006; Helmuth et al. 2006; Diaz and Rosenberg 2008; Hawkins et al. 2008; Deutsch et al. 2011).

## Methods

### Field temperature measurements

Variation in estimated body temperature (T_b_) of *B. glandula* across tidal heights was characterized at the rocky intertidal zone near California Polytechnic State University’s Research Pier (Cal Poly Pier) facility in Avila Beach, California, USA (35°10′42″N 120° 44′35″W) using operative temperature models (OTMs). To create OTMs, acorn barnacles with large shells (base diameter >20 mm) were collected from the Cal Poly Pier pilings on snorkel. The shells were cleaned of all tissue and dried. Opercular plates were superglued together in the aperture to prevent them from falling out after tissue removal. Once dry, an iButton (model: DS1922L) was affixed inside the shell using silicone glue and allowed to set for at least 24 h before deployment. Loggers were adhered to rocks using marine epoxy (Splash Zone 2-part epoxy) at the highest and lowest positions where *B. glandula* was found in the intertidal zone at our field site. We deployed a total of 6 OTMs at each tidal position (high and low) for each consecutive collection period (∼2 months each), though there were occasional data-logger losses due to extreme swell events. Each OTM collected a temperature reading (±0.5°C) every 30 min for up to 80 days at which point the entire unit was replaced with a new OTM. Temperature readings were recorded from January 28 to November 6, 2018.

### Field body size measurements

**Fig. 1:**
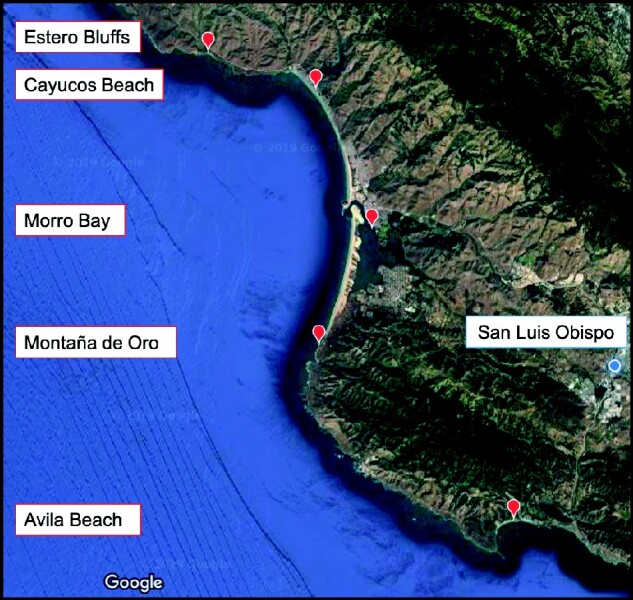
Map of rocky intertidal field sites where barnacle body size measurements were collected. Sites listed from north to south are as follows: Estero Bluffs State Park, Cayucos Beach, Morro Bay, Montaña de Oro State Park (Hazards Reef), Avila Beach (base of the Cal Poly Research Pier). Sites were sampled during low tide from June to August 2018. Measurements were collected *in situ* from barnacles adhered to rocks or mussels in the intertidal zone, except at the Morro Bay site where barnacles were located on pier pilings.

**Fig. 2:**
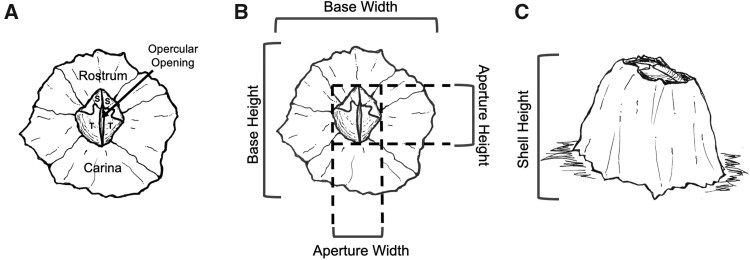
Barnacle external anatomy and body size metrics. (**A**) Barnacle shell anatomy: shell plates including rostrum, carina, and opercular plates (two scutal plates [S] and two tergal plates [T]) surrounding the opercular opening/slit. (**B**) Barnacle shell base and aperture metrics: base dimensions were measured at the base of the barnacle where the shell meets the substrate and aperture dimensions were measured at the edges of the opening at the top of the shell (i.e., the aperture) surrounding the opercular plates. Specifically, base and AH measurements were measured at the widest point along the carino-rostral axis, whereas base and AW measurements were measured at the widest point perpendicular to that same axis. (**C**) Barnacle SH metric: SH was measured from substrate to the tallest point of the barnacle shell. All measurements were made using digital calipers, except field measurements of SH, which were measured with a digital depth gage.

We quantified the average body size of *B. glandula* across their vertical distribution at five different field sites along the central coast (Fig. 1): Estero Bluffs State Park (35°27′21.7″N 120°57′47.7″W), Cayucos Beach (35°26′29.7″N 120°53′48.0″W), Morro Bay (35°22′15.1″N 120°51′29.0″W), Montaña de Oro State Park (Hazards Reef; 35°17′22.6″N 120°53′02.8″W), and Avila Beach at the base of the Cal Poly Pier (35°10′41.8″N 120°44′38.4″W). Body size metrics from individual barnacles were measured in the low, middle, and high intertidal positions from four perpendicular vertical transects laid down every 2 m along an 8-m horizontal transect. The single exception to this protocol was at the Morro Bay site where barnacles were measured on pier pilings. At this site, individual vertical transects were placed on separate pier pilings located within a few meters of each other. All measurement events occurred during low tide periods at a tidal height of ≤0.4 ft. We visually approximated the location of the highest and lowest *B. glandula* along each vertical transect. To establish the location of the middle intertidal barnacles, we measured the approximate distance (as length along the rock face) between the top- and bottom-most individuals of this species and measured barnacles at a distance exactly intermediate to the two. It should be noted that the lowest, middle, and highest positions of their distribution do not exactly correspond to the position of the high, middle, and low intertidal zones, but they are a very close approximation. As such, terms used to describe the intertidal zones (i.e., low, middle, and high) and relative barnacle position in the intertidal (i.e., lowest, middle, and highest) will be used interchangeably throughout this paper to represent intertidal position. At each intertidal position we placed a 5 × 5 cm quadrat immediately to the right side of each vertical transect. All individuals within each quadrat with an aperture diameter ≥2 mm were measured. Anything smaller was difficult to distinguish from other barnacle species and was considered too small to get an accurate measurement. If there were no barnacles in the initial quadrat placement, it was flipped to the right until at least one individual of the appropriate size was found within the quadrat. For each barnacle in the quadrat we measured the following metrics of body size: aperture width (AW; carina-rostral), aperture height (AH), base width (BW; carina-rostral), base height (BH), and shell height (SH) (Fig. 2). In some cases, not all measurements from a single individual could be made owing to barnacle clustering or awkward positioning within cracks in the rocks.

### Collection and maintenance of experimental barnacles

All barnacles used for lab experimentation in this study were collected from the rocky intertidal zone at the base of the Cal Poly Pier. At this location, we established a semi-permanent, 20 m horizontal transect in an area characterized by abundant *B. glandula*. Barnacles were collected from vertical transects laid down—in a perpendicular orientation—every 1 m along the 20 m horizontal transect. As in the field body size analysis, we visually approximated the location of the highest and lowest barnacles along each vertical transect, and estimated the exact midpoint for the middle barnacles. We then located barnacles for collection using the same quadrat (5 × 5 cm) sampling procedure (see section Field body size measurements), and removed barnacles from the highest, middle, and lowest intertidal positions. The largest few individuals (operculum diameter ≥2 mm) within each quadrat were collected. The exact number of barnacles collected within each quadrat varied by experiment (typically 1–3; see below). Since *B. glandula* does not form a solid calcified base, the substrate found under the barnacle, whether rock or shell of another invertebrate (typically mussels), would be removed with the barnacle. All sampling events occurred during low tide periods at a tidal height of ≤0.4 ft.

Barnacle collections for Experiment 1 (aquatic respirometry, behavior, and enzyme activity measurements) occurred between August 8 and October 24, 2018 and took place during low tides between 23:00 and 7:00 am. For these collection events, only a single vertical transect was sampled for high, mid, and low tidal heights each day (*n* = 1 barnacle collected per tidal height per day; *N* = 20 vertical transects sampled in total over 20 days). After collection, animals were transported in air back to a holding facility on the main campus. Here, barnacles were placed in 38 L aquaria with recirculating, filtered seawater (FSW; 14°C, 34 ppt) until 8:00 am that morning at which point they were removed for immediate experimentation.

Barnacle collections for Experiment 2 (aerial respirometry) occurred between June 30 and August 15, 2019 and took place during low tides between 4:00 and 8:00 am. As above, only a single vertical transect was sampled for high, mid, and low tidal heights each day (*n* = 1 barnacle collected per tidal height per day; *N* = 15 vertical transects sampled in total over 15 days). After collection, animals were transported and housed under the same conditions as above until 12:00 that morning, at which point they were removed for immediate experimentation.

Barnacle collections for Experiment 3 (aerial D-lactate measurements) occurred between December 15, 2020 and January 12, 2021, for a total of three sampling periods (*n* = 24 barnacles per tidal height per day). During each collection event six vertical transects were each sampled for high, mid, and low tidal heights (*n* = 4 barnacles per tidal height per transect). After collection, animals were immediately brought to the end of the adjacent research pier where we measured metrics of body size (body mass, AW, AH, BW, BH, and SH; Fig. 2) and tagged barnacles with identification numbers by affixing small, labeled latex squares with superglue to the base of each shell. Barnacles were then placed in a 38 L glass aquarium (“experimental tank”) and treated with aerated, flow-through, unfiltered seawater pumped directly from the ocean below (11.8–12.5°C, 33.2–33.5 ppt). Barnacles remained under these conditions for 18 h until experimentation, during which time they fed on plankton naturally present in the unfiltered seawater in which they were maintained.

### Experiment 1: Aquatic respirometry, behavior, and enzyme activity measurements

In our first experiment, we simultaneously measured O_2_ consumption rates and quantified behavioral activity patterns from the same subset of barnacles (*n* = 19–20 barnacles per tidal height) while they were immersed in FSW (at 14°C) within respirometry chambers. We later went on to process these individuals for whole-animal CS and lactate dehydrogenase activity. Our measurements of whole-animal O_2_ consumption rates serve as estimates of the routine metabolic rate (RMR) for individual *B. glandula.* RMR is the rate of energy use by an organism during typical, routine behavior. In the case of *B. glandula*, “typical behavior” will include some amount of opercular and cirral activity while the sessile barnacle remains anchored in flowing seawater. The behaviors we quantified almost exclusively entail opercular pumping and cirri extension behaviors aimed at food capture and respiration, both of which have clear links to metabolism. And finally, CS and LDH enzyme activity levels are common tissue-level indicators of overall aerobic and anaerobic metabolic capacity, respectively (e.g., Dickson et al. 1993).

#### Aquatic respirometry

We estimated RMRs (at 14°C) of *B. glandula* from high, middle, and low intertidal positions using an intermittent-flow respirometry system (Loligo Systems, AutoResp Software version 2.2) that determined O_2_ consumption rates (MO_2_; μmol O_2_/h*mg tissue) of individual barnacles immersed in 14°C FSW over a 24-h period. See Resner et al. (2020) for complete methodological details. In short, one barnacle from each tidal position (high, middle, and low) was randomly assigned to one of four glass respirometry chambers (21 mL) where it was placed base-side down in the center of the chamber. The fourth chamber was left empty to serve as a control to account for microbial respiration. These four chambers were then anchored, submerged, within a larger acrylic seawater bath (9 L), which was maintained at a constant temperature of 14°C with an aquarium chiller and bubbled with atmospheric air. This seawater pool served as both a temperature bath and the source of freshly oxygenated seawater with which to periodically flush the chambers.

Aquatic MO_2_ values were determined using an intermittent respirometry protocol that consisted of a repeating series of (1) a 5 min flush cycle, during which time a set of pumps were used to flush the chambers with fully oxygenated seawater from the surrounding water bath, (2) a 5 min wait period, when flush pumps were turned off and O_2_ levels were allowed to stabilize within the closed chambers, and (3) a 1 h measurement period, when the rate of pO_2_ decline in each chamber was actively measured to determine the MO_2_ resulting from respiration. pO_2_ values were measured with a fiber optic Witrox 4 sensor that measured changes in the fluorescence level emitted from an O_2_-sensitive “sensor spot” glued to the inside of each chamber. Throughout all three cycles an additional set of pumps maintained constant, recirculating seawater flow (∼13 cm^3^/s) within every individual chamber to ensure O_2_ homogeneity. At the start of each respirometry trial, barnacles experienced a single, complete intermittent cycle (flush, wait, and measurement; ∼1.3 h) prior to any data collection. Following this adjustment period, these three steps were repeated cyclically for 24 h, during which time MO_2_ measurements were made during every measurement period. The final MO_2_ value for each individual barnacle was an average of the MO_2_ values collected during each of these measurement periods (∼17 per trial). Obtaining data over a 24 h time period allowed us to account for any circadian rhythm effects on RMR. Further, each respirometry trial was initiated at approximately the same time each day (11:00 to12:00) to minimize variability.

MO_2_ values for each barnacle (and the empty control chamber) were derived from the change in O_2_ concentration within each chamber during each measurement period over the 24 h. The [O_2_] values (mg O_2_/L) were automatically calculated by the AutoResp software from the (1) sensor spot-determined O_2_ pO_2_ value and (2) the manually input values for seawater salinity (ppt), temperature (°C), and atmospheric pressure (mbar). Whole-animal O_2_ consumption rates (MO_2_; mg O_2_/h) were then calculated using the following equation:

(1) $$MO_{2}\;(\mathrm{mg}\;O_{2}/h)=\frac{{\left[O_{2}\right]}_{0}-{\left[O_{2}\right]}_{1}}{t}\cdot V$$

[O_2_]_0_ = O_2_ concentration at time 0 (mg O_2_/L)

[O_2_]_1_ = O_2_ concentration at time 1 (mg O_2_/L)

V = chamber volume minus volume of barnacle (L)

*t* = *t*_1—_*t*_0_ (h)

The whole-animal MO_2_ (mg O_2_/h) for each barnacle was then corrected by subtracting the MO_2_ of the empty control chamber from the MO_2_ of each barnacle chamber. Finally, mass-specific MO_2_ values (μmol O_2_/h*mg tissue) were calculated by dividing the whole-animal MO_2_ value by the wet weight (mg) of the barnacle following its complete tissue dissection from the shell, and converting mg O_2_ to μmol of O_2_ using the molar mass.

#### Behavior

During the second and third measurement period of each intermittent respirometry trial, we performed a real-time behavioral observation by visual inspection as follows. Starting immediately at the beginning of the second measurement period, we observed each of the three barnacles—in a randomly selected order—for 5 min each. We subsequently waited five additional minutes, with no observation, then repeated the 20 min observation cycle twice more. Thus, in a single respirometry measurement period, we observed each barnacle for a total of 15 min. Each round of observations would start with a different chamber, to ensure no one barnacle was recorded at the same time during each respective observation period. We then repeated the entire observation protocol during the third respirometry measurement period. In total, each individual barnacle was observed for 30 min.

During our observations, we determined the total amount of time each barnacle spent displaying the following behaviors: (1) operculum closed; (2) testing, operculum open with no cirral extension; (3) pumping beat, operculum open with cirral movement, but no unfurling; (4) normal cirri beat, operculum open with full cirri extensions and retractions; (5) fast cirri beat, operculum open with cirri extending and retracting quickly, though not completely back inside the shell; and (6) extension, cirri held extended without any retracting. This behavioral classification scheme is based on the detailed account of Crisp and Southward (1961) (see also Anderson 1981; Anderson and Southward 1987). Additionally, we measured the pumping beat frequency (PBF) and normal CBF. Rates for both variables were determined as the amount of time it took for the barnacle to extend and retract their cirri 5 times, and expressed as beats/second.

#### Enzyme activity

Immediately following their removal from the respirometry chambers, barnacle tissues in their entirety were rapidly dissected from the shell, flash-frozen in liquid nitrogen, and stored at −80°C until later enzyme activity analysis. We used standard spectrophotometric assays to quantify CS and lactate dehydrogenase activity in whole-animal tissue homogenates. Frozen tissues (5–125 mg) were homogenized via a motorized homogenizer in 175 µL of potassium phosphate buffer (50 mM KPO; pH 7.5) and centrifuged at 10,000*g* for 10 min at 4°C. The supernatant was separated into aliquots to prevent freeze-thaw cycles as we later carried out CS and LDH activity measures on the same samples. Aliquots were immediately flash-frozen in liquid nitrogen, and stored at −80°C until further analysis.

To determine CS activity, 10 µL of undiluted supernatant was added to 180 µL of assay buffer (0.2 mM DTNB, 0.3 mM Acetyl CoA; pH 8.2) in a 96-well plate. A background absorbance reading was obtained over 10 min at 412 nm (Victor X4 Multimode Plate Reader, Perkin Elmer) to ensure endogenous oxaloacetate was used up before measuring CS activity. After a stable background reading was obtained, the reaction was quickly initiated by the addition of 10 µL of oxaloacetate (final concentration 1 mM) to every well (final volume 200 µL). CS activity (μmol citrate/g tissue*min) was then determined from the change in absorbance at 412 nm over 5 min using an extinction coefficient for 5-thio-2-nitrobenzoic acid of 13.6 mM^−1 ^cm^−1^.

To determine LDH activity, 10 µL of undiluted supernatant was added to 170 µL of assay buffer (52.5 mM imidazole, 0.15 mM NADH; pH 7.5) in a 96-well plate. A background absorbance reading was obtained over 10 min at 340 nm to ensure endogenous pyruvate was used up before measuring LDH activity. After a stable background reading was obtained, the reaction was quickly initiated by the addition of 20 µL pyruvate (final concentration 2.64 mM) to every well (final volume 200 µL). LDH activity (μmol lactate/g tissue*min) was determined from the change in absorbance at 340 nm over 5 min using an extinction coefficient for NADH of 6.22 mM^−1 ^cm^−1^. All samples were run in triplicate for both CS and LDH activity measurements. All assays were run at a common temperature (20°C), which ensures valid statistical comparability between treatments and is an ecologically relevant temperature in the field.

### Experiment 2: Aerial respirometry

To complement the aquatic respirometry data generated in our first experiment, we carried out a separate experiment to measure O_2_ consumption rates of *B. glandula* during emersion at a common temperature (14°C). Aerial MO_2_ values were collected from 10 to 12 barnacles per tidal height using the same respirometry system that we used to gather aquatic MO_2_ values (see Aquatic respirometry section). The only differences with the protocol were that (1) the glass respirometry chambers were air-filled, not FSW-filled, (2) a non-intermittent, closed-system respirometry protocol was carried out over a single 24-h measurement period owing to the relatively slow decline of O_2_ in an air-filled chamber of similar volume to an FSW-filled chamber, and (3) there was no mechanism for mixing air within each closed chambers as this was deemed unnecessary in previous studies of aerial respiration in barnacles (Resner et al. 2020). To reduce chamber volume, and hence permit measurable rates of O_2_ consumption while in air, an approximately equal volume of small, sterilized glass beads (9 mL) was placed into each chamber (21 mL) alongside the barnacle (or the empty control) to serve as an inert space filler. As before, obtaining MO_2_ data over a 24-h time period allowed us to account for any circadian rhythm effects on RMR, and each respirometry trial (*N* = 12 trials, each trial containing a barnacle from every tidal height) was initiated at approximately the same time each day (approximately 12:00 to 13:00) to minimize variability.

Aerial, whole-animal MO_2_ (mg O_2_/h) values were calculated from Equation (1) and corrected for background O_2_ consumption from the empty chamber, as with the aquatic MO_2_ values. However, the aerial MO_2_ calculation required one additional conversion that was unnecessary in the aquatic MO_2_ calculation. The most current version of the AutoResp software version 2.2.2 assumes by default that measurements are being made in water (of a specifiable salinity, temperature, and atmospheric pressure)—not air—when it converts the sensor-determined pO2 values to [O_2_] values. This will grossly underestimate the change in O_2_ concentration in air with a drop in pO_2_, as the [O_2_] is ∼30 times greater in air than seawater at the same temperature and partial pressure. Thus, we multiplied our final MO_2_ values by a conversion factor of 33.9 (determined with the Ideal Gas Law), which represents the exact fold-difference in [O_2_] between the air and saltwater (34 ppt) at the same pO_2_ for a temperature of 14°C and an atmospheric pressure of 760 torr. Final mass-specific MO_2_ values (μmol O_2_/h*mg tissue) were then calculated, as before, by dividing the corrected, whole-animal MO_2_ by the wet weight of the barnacle’s entire tissue mass following dissection from the shell and converting from milligram to micromol with the molar mass of O_2_. For all barnacles, the consumption of O_2_ over time was linear, suggesting that O_2_ levels within each chamber never fell below the critical partial pressure (*P*_crit_) for this species.

### Experiment 3: D-Lactate measurements during air emersion

We measured whole-body [D-lactate] (mM) to assess the amount of anaerobic activity occurring during emersion in *B. glandula.* To do this, we collected barnacles from the high, middle, and low intertidal positions (see Collection and maintenance of experimental barnacles section) and exposed them to air for varying amounts of time (0, 6, 24, or 96 h). Barnacles were randomly assigned a position within a single 38 L glass aquaria (“experimental tank”) to ensure that any possible variation in ambient conditions (e.g., sunlight, temperature, and seawater flow) during the acclimation or exposure period would be experienced randomly between the treatment groups. After the 18-h acclimation, the tank was drained of seawater, and barnacles were left in the damp tank in air for up to 96 h. Over this exposure period, ambient conditions in the experimental tank were allowed to vary as they were in the natural environment to more closely approximate what the barnacles could experience *in situ* during air emersion (11.8–17.0°C; 68–91% relative humidity; ambient photoperiod). A subset of barnacles (*n* = 6 per tidal height per time point per sampling day) was dissected immediately after removal of the seawater (*t* = 0 h), and then another subset after 6, 24, and 96 h exposure to air. During dissections, all possible tissue was removed from the shell, flash-frozen in liquid nitrogen, and stored at −80°C. This entire process was carried out times.

Barnacles possess lactate dehydrogenases that are specific to D-lactate (Gleason et al. 1971; Ellington and Long 1978; Vial et al. 1999; López et al. 2003; Rangaswami et al. 2020), unlike most other crustaceans that produce L-lactate (Gleason et al. 1971; Gäde and Grieshaber 1986). As such, we quantified whole-animal D-lactate concentrations using basic spectrophotometric assays. Tissue samples (∼10–80 mg) were homogenized in 5 volumes of 10% trichloroacetic acid, centrifuged for 15 min at 10,000*g* at 4°C, and supernatants were stored at −80°C until further analysis. To quantify D-lactate, 16 µL of the supernatant was added to 210 µL of assay mixture (combined at a ratio of: 1 mL glycine buffer, 2 mL distilled water, 18 µL [50 U] D-LDH, 5 mg NAD^+^, 3.7 mg EDTA) and allowed to incubate for 45 min at room temperature. Endpoint absorbance values were measured at 340 nm for each sample (Victor X4 Multimode Plate Reader, Perkin Elmer). D-lactate levels (mM) were then determined by comparison to a standard curve generated from D-lactate samples of known concentration (0–5 mM) on each plate. All samples, standards, and blanks were run in duplicate. Final values for all samples and standards were corrected for background NADH absorbance from a sample of D-LDH-free assay buffer.

### Statistical analysis

All statistics were conducted in JMP software version 13 or RStudio version 1.1.442; R version 4.0.2. Data were tested for normality using the Shapiro–Wilk test, and for homogeneity of variance using Levene’s test. Data that did not meet the assumptions for normality were log-transformed (Log_10_ [variable]) and assumptions of normality and homoscedasticity were re-checked before use with parametric statistics. When log-transformations were unsuccessful at achieving normality and equal variance, non-parametric Kruskal–Wallis tests were used.

Principal component analyses (PCAs) were performed to determine associations among barnacle body size parameters within each separate data set. Principal components (PCs) with an eigenvalue >1 (or a percentage of total variance >50%) were retained for use as a composite variable of body size in subsequent statistical analyses. In all cases, there was only a single principle component that met this criterion, hereafter referred to as first PC (PC1) (e.g., Table 1). Where necessary in our statistical analyses, data were corrected for barnacle body size by using the PC1 variable (as opposed to simply tissue mass, SH, etc.).

**Table 1:** PCA results based on the five body size measurements (AH, AW, BH, BW, and SH) collected from barnacles in the field body size experiment

PC number	Eigenvalue	Percent	Size metric (mm)	PC1 factor loadings
1	4.1142	82.284	AH	0.905
2	0.3442	6.884	AW	0.898
3	0.2593	5.185	BH	0.931
4	0.1560	3.120	BW	0.912
5	0.1263	2.527	SH	0.887

Left: Top five PCs with their respective Eigenvalues and percentage of variation explained by each PC. Right: Factor loadings for PC1 from the PCA performed on the five body size metrics.

Linear regression models, including multiple regression (MR), ANOVA, and ANCOVA, were used for all models meeting assumptions of normality, linearity, independence, and homogeneity of variance. Negative binomial generalized linear models (NB-GLMs) with a log link function (GLM, MASS package, R; Venables and Ripley 2002) were used when MR assumptions were not met and data were overdispersed. Model assumptions for GLM models were assessed according to Zuur et al. 2009. Akaike Information Criterion (AIC) model selection was used to assess variable inclusions (i.e., tidal position, transect, or body size) for all MR and GLM models; models with the lowest AIC were selected in all cases (Δ ≥ 3 in all instances). Additional linear regression analyses were used to look for relationships between barnacle body size (as PC1 or AH) and each response variable of interest to further confirm the inclusion of a body size variable in each respective statistical model. Response (dependent) variables, explanatory (independent) variables, and the specific statistical models used to analyze each combination of variables can be found in Table 2. When there were significant effects in any of the statistical models, *posthoc* pairwise comparison tests were performed using Tukey’s Honestly Significant Difference (HSD) tests (parametric models) or Steel–Dwass tests (nonparametric models). Results were considered to be significant at the *α* = 0.05 level.

**Table 2:** Dependent variables, independent variables retained for analyses (as chosen by AIC model selection), and statistical models used for hypothesis testing

**Dependent variable**		Independent variable(s)			Statistical model
Body size	AH (mm)	Intertidal position	Site		MR
	PC1	Intertidal position	Site		MR
Enzyme activity	Size-adjusted CS activity^a^	Intertidal position	—		One-way ANOVA
	Size-adjusted LDH activity^a^	Intertidal position	—		One-way ANOVA
Respirometry	Aquatic MO_2_ (μmol O^2^/h·mg) ^a^	Intertidal position	—		MR
	Aerial MO_2_ (μmol O^2^/h·mg) ^a^	Intertidal position	Transect		MR
Behavior	Total time active (s) ^a^	Intertidal position	Transect		NB-GLM
	Time closed (s)	Intertidal position	—		Kruskal–Wallis
	Time testing (s)	Intertidal position	—		Kruskal–Wallis
	Time pumping (s)	Intertidal position	—		Kruskal–Wallis
	Time normal beating (s)	Intertidal position	—		Kruskal–Wallis
	Time fast beating (s)	Intertidal position	—		Kruskal–Wallis
	PBF (beats/s)	Intertidal position	PC1^b^		ANCOVA
	CBF (beats/s)	Intertidal position	PC1^b^		ANCOVA
Lactate	[D-lactate] (mM) ^a^	Intertidal position	Time	PC1^b^	Two-way ANCOVA

*Note:* Body-size adjusted CS and LDH activity data were generated by dividing raw activity values (μmol/g·min) by PC1 (dimensionless) before use in statistical analyses. Mass-specific MO_2_ values were used in all statistical analyses. ^a^Dependent variables annotated were log_10_-transformed prior to analysis. ^b^Independent variables annotated served as the covariate in the respective ANCOVA model.

## Results

### Field temperature measurements

**Fig. 3:**
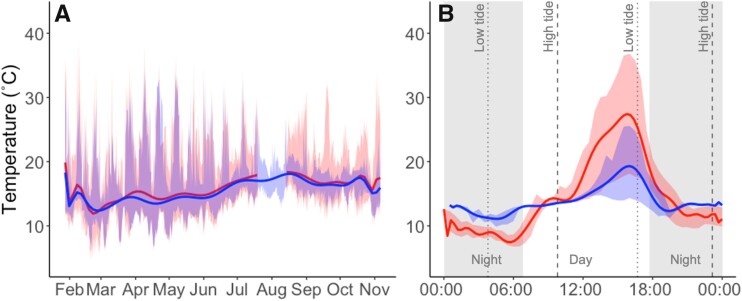
Field temperature measurements from the high (red) and low (blue) intertidal positions of *B. glandula* at the base of the Cal Poly Pier (Avila Beach) over the course of a single year (**A**) and single day (**B**). OTMs were anchored to rocks in the intertidal zone within the typical highest and lowest regions of the *B. glandula* vertical distribution at this site (*n* = 6 OTM/tidal position) where they recorded a temperature measurement every 30 min for the entire deployment period. (**A**) Daily mean temperature (bold lines) and maximum/minimum temperatures (shaded region) for ∼ 1 year from January 2018 to November 2018. (Heavy wave action led to the loss of all six OTMs at the high position for a small portion of late summer, which accounts for the lack of high intertidal data around August.) (**B**) Hourly mean temperature (bold lines) and maximum/minimum temperatures (blue and red shaded regions) for a single 24-h day (February 16, 2018). This graph serves to more finely resolve temperature variation associated with the tidal cycle. Vertical dashed lines represent the time of the daily high tides (red) and low tides (blue), and the gray shaded regions represent nighttime periods.

Using OTMs to approximate body temperature (T_b_) of barnacles across the intertidal zone, we confirmed that temperature varied across both annual (Fig. 3A) and diel (Fig. 3B) cycles. Annual temperature variation can be explained by seasonal differences (e.g., summer versus winter temperatures), whereas daily temperature fluctuations are driven by the tidal cycle. As predicted, the maximum and minimum daily temperatures tended to be more extreme in the high intertidal positions compared to the low, despite the fact that mean T_b_ was ∼15°C across tidal heights (Table 3). We found that over the course of nearly a year, OTMs in the high intertidal zone were >4 times more likely to experience temperatures above 30°C than those in the low intertidal, and were the only OTMs to experience temperatures >40°C (no OTMs in the low intertidal measured temperatures >40°C; Table 3). That said, barnacles in the high tidal position still spent ∼99% of the time at temperatures 30°C.

**Table 3:** Summary of extreme temperature (°C) conditions in the intertidal zone at the base of the Cal Poly Pier (Avila Beach) as measured by the OTMs anchored at the typical high and low tidal height positions of *B. glandula* at this site over the course of ∼1 year (2018)

	Temperature (°C)				
Tidal height	Max	Min	Mean	Percent time above 30°C	Percent time above 40°C
High	42	4.5	15.5 ±4.4	1.179	0.026
Low	38	5.6	15.3 ±2.9	0.279	0.000

The max and min values represent the single highest and single lowest recorded temperatures of any OTM in each respective intertidal position over the entire measurement period. The mean is the average temperature from each position recorded across the entire year. Note that even though the mean temperature is very similar between the high and low tide positions, the max and min temperatures are more extreme in the high intertidal.

### Field body size measurements

**Fig. 4:**
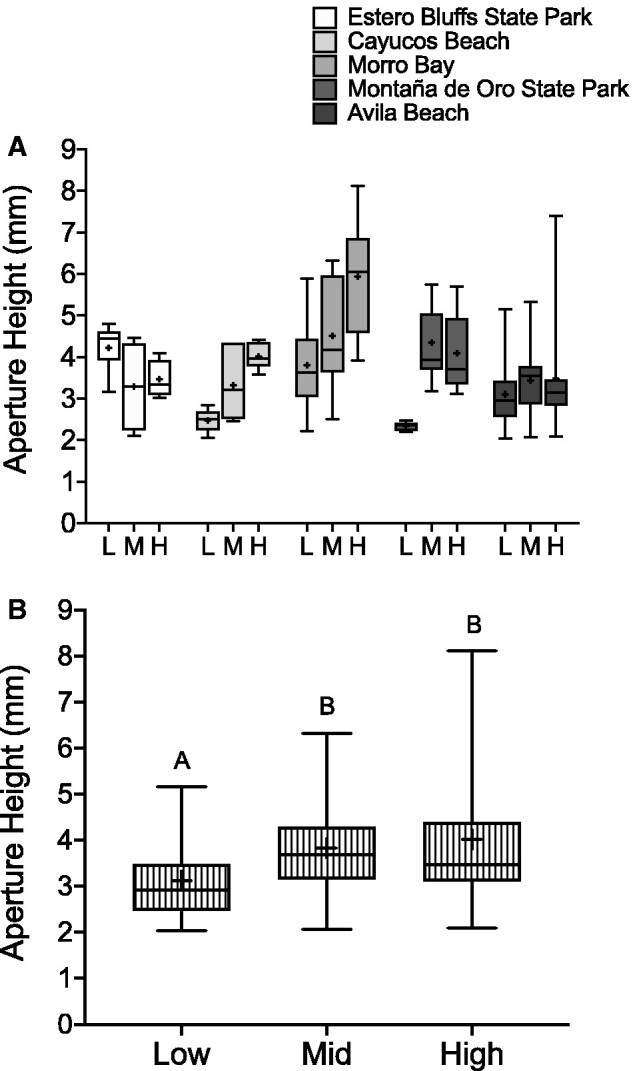
Differences in AH (mm) of *B. glandula* residing in each intertidal position (low, middle, and high) at several field sites along the central coast of California. Field measurements of *B. glandula* AH displayed by (**A**) site and (**B**) pooled across sites. In both figures, the whiskers represent the min and max values, the box extends from 25th to 75th percentiles, the line represents the median, and the “+” indicates the mean. Columns with different letters are significantly different from one another (Tukey’s HSD, *P* < 0.05).

We measured five morphometric variables to estimate barnacle body size in the field (Fig. 2), and found that all body size measurements were highly correlated (*r* = 0.73–0.87; all P < 0.001). A PCA of all body size metric values collected from barnacles in the high and low intertidal revealed that the PC1 accounted for 82.3% of the variation (*λ* = 4.114), whereas all subsequent PC values were <7%, and the factor loading values indicated that all morphometric variables loaded positively on PC1 (Table 1). Thus, we performed MR analyses of the effect of tidal position on barnacle body size using the composite variable PC1. Additionally, we performed a replicate MR analysis with AH (Fig. 4) as our dependent variable, owing to the fact that AH had the greatest sample size and achieved the most statistical power.

The MR model revealed that there was a significant effect of tidal position on PC1 after accounting for the effect of sampling site (*F*_6,140_ = 7.33, *P* < 0.001), whereby barnacles from the low intertidal were significantly smaller than barnacles from the high intertidal (data not shown). Using AH as the response variable, rather than PC1, resulted in the same general model output (*F*_9,174_ = 5.842, *P*-value < 0.0001; Fig. 4). Barnacles in the low intertidal had an AH that was on average 20% smaller than those in the high intertidal (Fig. 4B).

### Enzyme activity, respirometry, and behavioral measurements

#### Enzyme activity

**Fig. 5:**
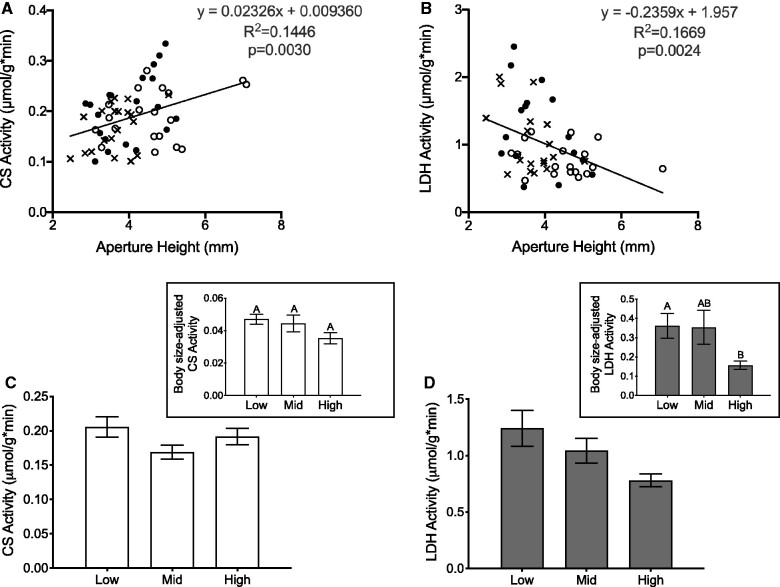
Effects of barnacle body size (**A and B**) and intertidal position (**C and D** and insets) on CS (left) and lactate dehydrogenase (right) enzyme activity (μmol/g*min) in *B. glandula*. (**A and B**) Relationship between barnacle shell AH (mm) and CS (A) or LDH (B) enzyme activity in *B. glandula* from low (filled circle), mid (cross symbol), and high (open circle) intertidal positions. There was a significant positive relationship between AH and CS activity, and a significant negative relationship between body size and LDH activity. The same significant relationships were observed if body size was represented by PC1, rather than AH (data not shown). Note for both enzymes the large degree of overlap in the range of body sizes of the barnacles collected from each intertidal position. In each figure the solid back line represents the linear regression line of all data pooled across tidal positions. (**C and D** and insets) Effect of intertidal position (low, middle, and high) on raw, whole-animal CS (white; **C**) and LDH (gray; **D**) activity values and on the body size-adjusted CS and LSH activity values (raw activity value divided by body size PC1) on which the respective statistical analyses were based (inset graphs). Columns with different letters are significantly different from one another (Tukey’s HSD; *α* = 0.05). All values represent means ± SEM; *n* = 16–20 barnacles per intertidal position.

Linear regression analyses revealed that there was a positive relationship between CS activity and barnacle body size (AH: *F*_1, 57_ = 9.63, *P* = 0.0030, *R*^2^ = 0.14; PC1: *F*_1, 57_ = 18.16, *P* < 0.0001, *R*^2^ = 0.24) (Fig. 5A) and a negative relationship between LDH activity and barnacle body size (AH: *F*_1, 51_ = 10.22, *P* = 0.0024, *R*^2^ = 0.17; PC1: *F*_1, 53_ = 20.40, *P* < 0.0001, *R*^2^ = 0.29) (Fig. 5B), as a result of which body size was taken into account in all subsequent analyses of the effects of tidal height on enzyme activity. We found that there was no significant effect of tidal height on body-size adjusted CS activity (one-way ANOVA: *F*_2, 56_ = 2.20, *P* = 0.1205) (Fig. 5C and inset), but there was a significant effect of tidal position on body size-adjusted LDH activity (one-way ANOVA: *F*_2, 50_ = 4.57, *P* = 0.0151), whereby barnacles from low intertidal positions had greater LDH activity compared to those in the high intertidal zone (Tukey’s HSD, *P* = 0.0189) (Fig. 5D and inset).

#### Respirometry

**Fig. 6:**
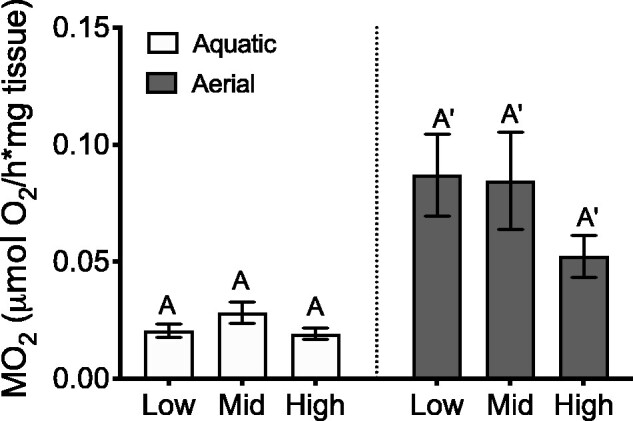
Effect of intertidal position (low, middle, and high) on whole-animal aquatic (white; *n* = 15–16 barnacles per intertidal position) and aerial (gray; *n* = 10–12 barnacles per intertidal position) O_2_ consumption rates (MO_2_; μmol O_2_/h*mg dissected body tissue) in *B. glandula* held at 14°C. Columns with different letters are significantly different from one another (Tukey’s HSD; *α* = 0.05). Values represent means ± SEM.

Using an MR model that incorporated transect, we determined that there was no significant main effect of tidal position on mass-specific O_2_ consumption rates of barnacles immersed in seawater (*F*_2,44_ = 1.522, *P* = 0.2295) or barnacles exposed to air (*F*_2,30_ = 1.8, *P* = 0.1821) (Fig. 6). If we also incorporated body size (as PC1) into the MR model, there were still no significant effects of tidal position on MO_2_ in air or seawater. O_2_ consumption rates were approximately 3–4 times greater in air compared to water across all treatments.

#### Behavior

**Fig. 7:**
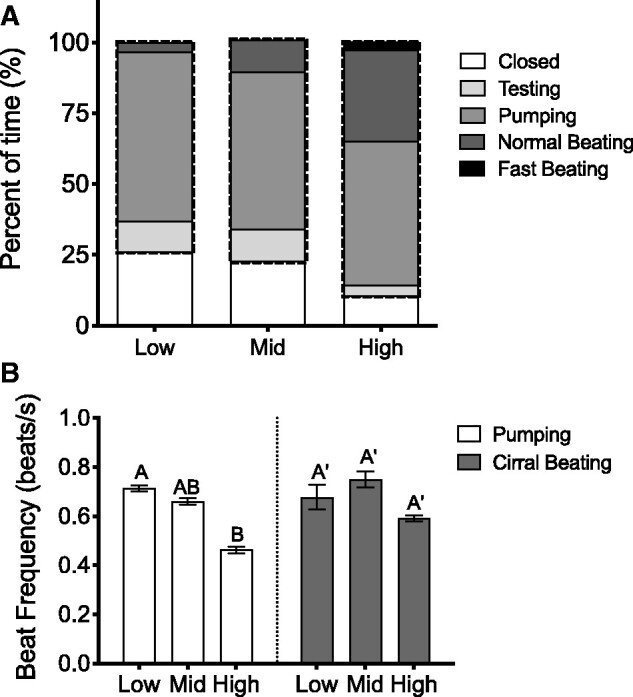
Effect of intertidal position (low, middle, and high) on behavior in *B. glandula.* (**A**) Average percentage of time barnacles from each intertidal position spent performing specified behavioral activities (closed, testing, pumping, normal cirral beating, and fast cirral beating) (*n* = 17–19 barnacles per intertidal position) over a 30 min measurement period. The dashed outline indicates the total percentage of time active; that is, the total percentage of time barnacles from each intertidal position spent with the operculum open and actively engaged in testing, pumping, normal beating, or fast beating. (*Note*: graphs indicate percentage of time [out of 30 min] that barnacles spent engaged in each behavior, whereas statistical analyses of the data in (A) were performed on whole number counts of time in seconds [out of 30 min] that barnacles spent engaged in each behavior.) (**B**) Average PBF (white) and cirral beat frequency (normal beat only; gray) of barnacles from each intertidal position (*n* = 15–19 barnacles per intertidal position). Note that measurements of beat frequency were only obtained from barnacles that actually exhibited each particular behavior during the observation period. Columns with different letters are significantly different from one another (Tukey’s HSD; *α* = 0.05). Values represent means ± SEM.

We found no significant relationship between body size (as PC1) and the time (in seconds) barnacles spent engaged in any behavior (linear regression analyses; closed, *P* = 0.44; testing, *P* = 0.66; pumping, *P* = 0.56; normal beating, *P* = 0.23; fast beating, *P *= 0.37; active, *P* = 0.41; data not shown). A negative binomial GLM revealed that intertidal position (and transect) had a significant effect on the total time barnacles were active (i.e., operculum open and engaged in either testing, pumping, or cirral beating). *Balanusglandula* from high intertidal positions spent significantly more time active than barnacles from the mid intertidal (*Z*_2,54_* *=* *−28.32, *P* < 0.0001) and the low intertidal (*Z*_2,54_* *=* *−34.56, *P* < 0.0001), and barnacles from the mid intertidal spent significantly more time active than barnacles from the low (*Z*_2,54_* *=* *−6.35, *P*< 0.0001) (Fig. 7A). After accounting for the effect of transect, barnacles from the high tide position engaged in 23% more total time with their operculum open and active (testing, pumping, normal beating, or fast beating) than barnacles from the mid tide position (95% CI* *=* *21–24%) and 27% more total time than barnacles from low tide positions (CI* *=* *26–28%).

The increase in total time active in the high intertidal barnacles was largely the result of a significant increase in the amount of time spent normal cirri beating (Kruskal–Wallis: *χ*^2^* *=* *12.31, df = 2, *P* = 0.0021), and presumably, a greater amount of time feeding while submerged, compared to barnacles from the low intertidal. Additionally, high intertidal barnacles were found to spend significantly less time testing than barnacles from the mid and low intertidal (Kruskal–Wallis; *χ*^2^=8.07, *P* = 0.0178). Barnacles from different tidal positions did not demonstrate any significant differences in the amount of time they spent engaged in any other “active” behaviors (e.g., pumping or fast cirral beating). Only one individual was observed to carry out a true extension behavior and so this particular behavior was excluded from all analyses.

Linear regression analyses reveled a significant negative relationship between body size and both PBF (PC1: *F*_1,49_* *=* *5.06, *P* = 0.0290; AH: *F*_1,49_* *=* *5.93, *P* = 0.0186) and CBF (PC1: *F*_1,24_* *=* *12.97, *P* = 0.0014; AH: *F*_1,24_* *=* *9.77, *P* = 0.0046) (data not shown). After adjusting for size, barnacles from the high intertidal were found to have slower pumping beat frequencies than those from low intertidal zones (ANCOVA with PC1 as covariate; *F*_2,47_* *=* *3.89, *P* = 0.0273), although there was no significant difference in the normal cirral beat frequency of barnacles from different tidal heights (ANCOVA with PC1 as covariate; *F*_2,22_* *=* *0.8433, *P* = 0.4437) (Fig. 7B).

### Lactate measurements during emersion

**Fig. 8:**
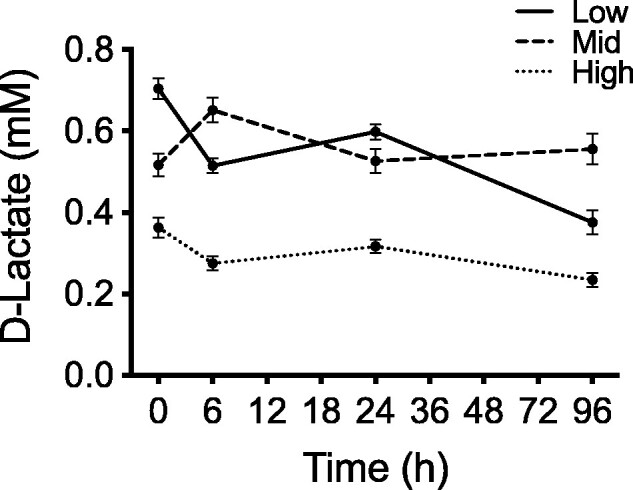
Effect of intertidal position (low, middle, and high) on whole-animal D-lactate concentration (mM) in *B. glandula* during air exposure. Average D-lactate concentration in barnacles from each relative tidal position following 0, 6, 24, and 96 h of air emersion (*n* = 15–18 barnacles per time point at each intertidal position). There was no significant accumulation of D-lactate over time in barnacles from any tidal height, though lactate levels were consistently lowest in the high intertidal barnacles. Values represent means ± SEM.

A two-way ANCOVA model (with body size PC1 as a covariate) revealed that there were no significant effects of time (D-lactate: *P* = 0.20) or the time*tidal height interaction (D-lactate *P* = 0.66) on log-transformed D-lactate concentrations. However, there was a moderately significant effect of tidal position on D-lactate (*F*_2,185_* *=* *2.98, *P* = 0.0532), whereby barnacles from the high intertidal position had a tendency toward the lowest lactate levels at all time points (Fig. 8).

## Discussion

An organisms’ vertical position in the intertidal zone (i.e., high versus low) affects the duration of time they spend cyclically emersed in air during low tide. Therefore, differences in vertical position result in predictable differences in the severity and degree of variability of the stressors they experience. Many of these stressors—particularly temperature, O_2_, and food availability—have direct and substantial impacts on metabolic rates in ectotherms. As such, we predicted that there would be differences in the metabolic phenotype between conspecifics in the low versus high intertidal. We found some evidence of tidal position-dependent differences in *B. glandula* metabolic phenotype. Specifically, we observed that barnacles from the low intertidal tend to beat their cirri less often when submerged, have a greater capacity for anaerobic metabolism (as indicated by increased baseline LDH activity and increased [D-lactate]) and be smaller in body size compared to conspecifics in the high intertidal. However, there were no significant differences in aerobic parameters like CS activity or baseline O_2_ consumption rates (in air or seawater) across tidal heights.

### Environmental variation across the intertidal

Our initial hypothesis rests on the assumption that there is sufficient variation in parameters that influence metabolism between the highest and lowest regions of the *B. glandula* vertical distribution to induce metabolic plasticity—or alternately drive selection. O_2_ levels are inherently stable in the air and in the well-mixed surface layer of seawater, and intertidal barnacles are typically very effective at aerial O_2_ uptake (Grainger and Newell 1965; Augenfeld 1967; Petersen et al. 1974; Vial et al. 1999; Davenport and Irwin 2003). Thus, hemolymph pO_2_ levels are not predicted to drop in *B. glandula* during emersion, or vary substantially between individuals in different tidal zones. If episodes of coastal hypoxia are frequent, however, barnacles residing in the lower intertidal could be subject to longer periods of reduced O_2_ levels than those in the high.

Where we do expect to see substantial variation between intertidal positions is in food availability, predation pressure, and especially temperature. Differences in food availability can be presumed without *in situ* measurements, owing to the fact that *B. glandula* are filter feeders and can only eat when submerged. Individuals anchored in the high intertidal, therefore, have less access to food due to increased time in the air. Differences in predation pressure can also be assumed given that the dominant predators of *B. glandula* are dogwhelks (*Nucella spp.*), sea stars, and the barnacle eating dorid (*Onchidoris bilamellata*), all of which predominantly feed in lower intertidal zones while submerged to avoid the physiological stress associated with prolonged emersion (Connell 1970; Bertness and Schneider 1976; Menge 1978; Dahlhoff et al. 2001; Yamane and Gilman 2009). Marine invertebrates, including *B. glandula*, commonly close their shell or operculum to avoid predation when chemical cues in the seawater suggest predators are present (Palmer et al. 1982; Reimer et al. 1995), and this might lead to internal hypoxia. Barnacles from the low intertidal may then be hypoxic more often than barnacles from the high intertidal given the increased amount of time they spend underwater in proximity to predators.

We used OTMs to examine the variation in body temperature profiles of *B. glandula* between their high and low vertical distributions at our field site. Over the course of ∼1* *year we observed that the mean T_b_ was virtually identical between barnacle OTMs in the high and low intertidal positions (∼15°C), but OTMs in the high intertidal position recorded greater temperature extremes (i.e., higher high and lower low T_b_) and more prolonged exposure to these extremes compared to those in the low intertidal positions (Fig. 3; Table 3). These patterns can be explained by more prolonged periods of air emersion in the high intertidal, and are consistent with other studies (Helmuth and Hofmann 2001; Zippay and Helmuth 2012; Miller and Dowd 2017). Exposure to air during low tide can lead to much warmer (e.g., if occurring during the day in the summer) or much colder (e.g., if occurring during the night in winter) temperatures than exposure to constant immersion because of the high degree of variation in air temperature compared to relatively stable sea-surface temperature.

The observed temperature variation between tidal heights, in combination with presumed differences in food availability and predation pressure, could serve to induce tidal position-dependent differences in *B. glandula* morphology and physiology—particularly in traits associated with metabolism.

### Body size distribution

Barnacle body size represents one such morphological measurement related to metabolism. Therefore, we initially performed an assessment of the relationship between intertidal position and average body size of *B. glandula* at several sites along the central California coast. We determined that almost invariably across different intertidal sampling locations on our coast, the largest individuals of *B. glandula* were found in the mid to upper intertidal zone, with smaller individuals occupying the lowest region of their vertical distribution (Fig. 4).

Variability in a species’ size distribution across the rocky intertidal zone is typically driven by either abiotic stress from emersion (e.g., desiccation and heat) or biotic stress during immersion (e.g., predation, competition for space, and lack of food). Many studies report that the largest individuals are found in the low intertidal (e.g., mussels (Dehnel 1956; McQuaid et al. 2000), chitons (Otaíza and Santelices 1985), and snails (Brown and Quinn 1988)), which is attributed to increased food availability and decreased stress during more prolonged periods of immersion. Likewise, Wethey (1983) observed that among the acorn barnacle *Balanus balanoides*, larger individuals were found in the mid intertidal compared to the high intertidal. These results collectively contradict our own. Several other studies, however, suggest that extreme abiotic stress, frequently desiccation stress, in the high intertidal selects against slow-growing, smaller individuals early on in settlement (Vermeij 1972; McQuaid 1982). Smaller individuals have a greater surface area:volume ratio (SA:V), which makes them less tolerant of desiccation stress. This can result in adults of larger body sizes in the high intertidal compared to those of a similar age in the low (e.g., limpets (Vermeij 1972); snails (McQuaid 1982)), as we observed herein.

We suggest that the observed effects of tidal position on *B. glandula* body size may be the combined result of several factors. First, as just mentioned, larger adults may dominate in the upper intertidal because thermal and desiccation stress impede survivability of small individuals to a greater extent than large individuals owing to their low SA:V. Another factor that seems certain to have contributed to our observation that smaller barnacles are more dominant in the low intertidal is the decreased amount of time they spend cirral beating—and therefore presumably feeding—when submerged compared to higher intertidal conspecifics (Fig. 7). Gillmor (1982) found that species of mussels characteristic of the high intertidal were not only better at acquiring energy via more frequent filter-feeding during immersion, but also more preferentially directed those energetic resources toward growth compared to other mussel species in the low intertidal. Predation and competition dynamics may also influence body size distributions. Common predatory whelks have been shown to prefer larger barnacles over small barnacles (Connell 1970; Lively et al. 2000). Given that their predation efforts are greater in the low intertidal zone, this could explain why we see an increased prevalence of large individuals of *B. glandula* in the more physiologically stressful high intertidal zone.

**Fig. 9:**
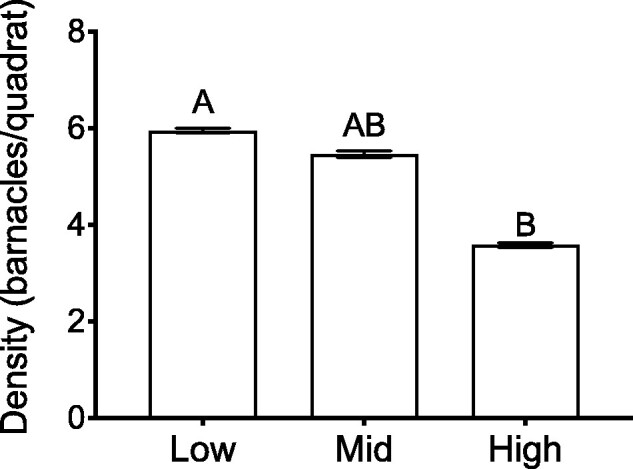
Estimate of average *B. glandula* density (no. of live barnacles/quadrat) at each intertidal position. Data are derived from the body size field analysis, and represent values pooled from all five field sites examined as the trend was the same at all sites. A one-way ANOVA revealed a significant effect of tidal position on barnacle density (*F*_2,166_ = 5.75, *P* = 0.0038). Columns with different letters are significantly different from one another (Tukey’s HSD; *α* = 0.05). All values represent means ± SEM; *n* = 53–64 quadrats per intertidal position.

There is also a well-known, though highly debated, inverse relationship between body size and population density (Damuth 1981). This relationship has held for certain temperate (Marquet et al. 1990) and tropical intertidal communities experiencing low predation pressure (Navarrete and Menge 1997), as well as certain barnacle species (Wethey 1983). Though this study was not directly aimed at measuring barnacle density in the field, we can estimate a crude measure of density at each tidal position from the total number of living barnacles in each systematically placed quadrat we examined in the field body size analysis. From these data, we observed that barnacle density is significantly lower in the high intertidal compared to the low intertidal positions (Fig. 9). Thus, decreased competition for space or resources due to decreased barnacle density in the high intertidal could partially explain the larger average body size here relative to the low intertidal.

A final driver for the size distribution pattern we observed could be the relatively high anaerobic capacity, and presumably increased anaerobic activity, characteristic of the lower intertidal barnacles (Fig. 5) that could come at the cost of growth. Organisms that are exposed to chronic hypoxia frequently utilize anaerobic metabolism and often have reduced growth rates compared to organisms not experiencing environmental O_2_ limitation (Wu 2002). We hypothesize that that same may be true for sessile invertebrates exposed to high predator densities or frequent coastal hypoxia. In the face of increased predation attempts, sessile invertebrates often keep their shell or operculum closed for prolonged periods of time and can experience hypoxia (Vial et al. 1992). In regards to hypoxia, local embayments on the California Coast, like San Luis Obispo Bay and Monterey Bay, are thought to be hotspots for upwelling and eutrophication-driven algal blooms that biologically induce nearshore hypoxia events. At our collection site in San Luis Obispo Bay at least six multi-day hypoxic events—defined as periods when the dissolved O_2_ levels of nearshore bottom water fell 4.6 mg L^−1^ hypoxic threshold suggested by Vaquer-Sunyer and Duarte (2008)—were reported between April and August of 2017 alone (R. Walter, personal communication). It is, therefore, likely that the barnacles used in this study were challenged by relatively frequent coastal hypoxic events. In combination with greater incidences of predation attempts, barnacles in the low intertidal that are submerged for a greater proportion of the time might, therefore, have reduced growth rates due to an increased reliance on anaerobic metabolism.

### Anaerobic metabolism

To quantitatively assess anaerobic capacity of adult *B. glandula* across their vertical distribution we measured their baseline LDH activity. From this, we determined that individuals from the low intertidal had significantly higher LDH activity, and therefore a greater anaerobic capacity, than barnacles from the high intertidal (Fig. 5D and inset). These data are consistent with previous works that collectively observed lower intertidal barnacle species rely more heavily on anaerobic metabolism during emersion than species adapted to higher intertidal positions, which rely principally on aerobic respiration in air (for review, see Vial et al. 1999). For example, Augenfeld (1967) found that *Chthamalus fossus* and *B. glandula*, species that dominate in the high intertidal and have, therefore, adapted to longer periods of air exposure, have much slower rates of glycogen utilization during prolonged emersion than *Tetraclita squamosa* and *B. tinntinnabulum*, species characteristic of the lower intertidal. From this, the authors surmised that the two upper intertidal species exploit mostly aerobic respiration in air, whereas the lower intertidal species were utilizing both aerobic and anaerobic metabolism. Simpfendörfer et al. (1995) also observed that aerial respiration rates were much closer to immersion respiration rates in upper intertidal barnacles (*Jehlius cirratus*; 80–100% of immersed O_2_ consumption rate) when compared to lower intertidal mussels (*Mytilus spp;* 5–50% of immersed O_2_ consumption rate), which again suggests the tendency toward fully aerobic respiration in air for high tidal zone species and anaerobic supplementation during air exposure in lower intertidal organisms. The LDH trends we observed in *B. glandula* are also consistent with our D-lactate findings. We found that low and mid intertidal *B. glandula* seem to have universally higher lactate concentrations relative to conspecifics from the high intertidal (Fig. 8); and this was true for both control barnacles (time 0 h) and across 4* *days of air emersion (time 6–96 h).

While we did see evidence of differences in baseline D-lactate levels in *B. glandula* based on tidal position, we did not find any significant accumulation of D-lactate during the 96 h of exposure to emersion (RH* *≥* *68%) in barnacles collected from any tidal position (Fig. 8). Rangaswami et al. (2020) likewise failed to observe an accumulation of D-lactate in air-exposed *B. glandula* (unless emersion was coincident with elevated temperatures, which we did not test in our study). Barnacles from the intertidal zone have adapted to prolonged air exposure by altering their behavior so as to maximize O_2_ uptake in the air and sustain aerobic respiration while preventing desiccation (Barnes and Barnes 1957; Barnes et al. 1963; Grainger and Newell 1965; Vial et al. 1999; Davenport and Irwin 2003). This is accomplished by the expulsion of internal seawater stores and the subsequent, repeated pulsation of a minute aperture in the tissue surrounding the opercular valves—a “pneumostome”—which gives the mantle cavity space surrounding the moist respiratory surfaces (e.g., gill-like branchiae, mantle tissues, and prosoma) access to O_2_-rich atmospheric air. It has been reported in the intertidal barnacle *Semibalanus balanoides* that during air emersion, a greater percentage of individuals in the low intertidal have their pneumostome open compared to those in the high intertidal, which further reflects their need to balance access to O_2_ with water conservation (Grainger and Newell 1965). We predict that lactate would begin to accumulate in *B. glandula* only after a period of many days in air (e.g., 6–10 days) when more permanent pneumostome closure becomes necessary to prevent desiccation, or during emersion episodes concurrent with very low humidity conditions and/or elevated temperatures (e.g., Barnes et al. 1963; Rangaswami et al. 2020).

These data then beg the question, why do low intertidal barnacles have an increased capacity for anaerobic metabolism? This seems counterintuitive given that they form pneumostomes when in air and likely keep them open more often than conspecifics in the high intertidal (Grainger and Newell 1965). We suspect that the driver for increased anaerobic capacity in low intertidal *B. glandula* may be events other than short-term air exposure. Gilman et al. (2013) showed that complete opercular closure was the only event that led to decreased metabolic rates for *B. glandula* while in water. When barnacles completely close their opercula, the O_2_ level of their internal mantle cavity fluid decreases rapidly, which over time can lead to increased anaerobic metabolism (Davenport and Irwin 2003). Thus, increased occurrences of events that elicit operculum closure could have the effect of inducing increased anaerobic capacity in barnacles. Under immersion conditions, barnacles will close their valves in response to coastal hypoxia (Barnes et al. 1963), decreased salinity (Davenport and Irwin 2003), and increased predator activity (Palmer et al. 1982). As mentioned previously, coastal hypoxia events occur with some regularity at our collection site in San Luis Obispo Bay (R. Walter, personal communication) and could potentially induce metabolic plasticity across the intertidal zone for our barnacles. We also know that most barnacle predators (e.g., asteroids and gastropods) rely on chemical cues to find their prey items while they are submerged (Kohn 1961; Castilla and Crisp 1970; Pratt 1974). Chemical cues are typically released by barnacles during cirral beating, therefore keeping their valves closed while immersed is one way to avoid predator detection (Barnes 2002). Palmer et al. (1982) have shown that during immersion, *B. glandula* remain tightly closed for extended periods of time when placed in contact with predatory stimuli (e.g., carnivorous snails and sea stars) compared to nonpredatory stimuli (e.g., algae, herbivorous snails, and sea stars). Naturally, low intertidal individuals will experience more closure-inducing events like hypoxia and predation given that they spend a longer duration submerged than those in the upper intertidal. Difference in the severity and exposure time for these events, rather than air emersion, may play a bigger role in driving the differences in anaerobic capacity of *B. glandula* across the intertidal.

### Aerobic metabolism

Given that we see greater anaerobic capacity in *B. glandula* from the low intertidal positions, we expected there to be greater aerobic capacity in conspecifics from the high intertidal. To assess aerobic capacity of *B. glandula* across their vertical distribution we (1) quantified baseline CS activity, an enzyme of the Krebs cycle that is used as a standard marker for aerobic capacity and (2) measured aquatic and aerial O_2_ consumption rates to determine RMR (14°C) under both conditions. In previous work, Augenfeld (1967) reported that barnacle species from the upper intertidal (*C. fossus* and *B. glandula*) had cytochrome oxidase activity levels (another enzymatic marker of aerobic capacity) and O_2_ consumption rates that were more than twice those from low intertidal and subtidal species (*T. squamosa, B. tinntinnabulum*, and *B. nubilus)*. Stillman and Somero (1996) also found that high intertidal porcelain crabs were able to maintain greater rates of respiration at a common temperature than those from the low intertidal zone. Despite these compelling data, we did not reveal any significant differences in CS activity (Fig. 5) or MO_2_ (aquatic or aerial) (Fig. 6) between conspecifics of *B. glandula* anchored at different tidal heights.

We know that limitations to aerobic performance are strongly implicated as a factor in determining thermal tolerance limits (Somero 2010). The O_2_- and capacity-limited thermal tolerance (OCLTT) concept, though not universally accepted (Jutfelt et al. 2018), postulates that inadequate capacity for the delivery of O_2_ to the tissues during temperature stress is the central physiological mechanism underlying species’ thermal tolerance ranges and resulting biogeographical distribution (see Pörtner 2010; Pörtner et al. 2017; Pörtner 2021). Several studies have also shown that intertidal organisms are living at temperatures very close to their thermal limits and as a result, may have no further capacity to tolerate temperature stress via acclimation (Tomanek and Somero 1999; Stillman and Somero 2000; Stillman 2003). So it may be possible that—by virtue of residing in the intertidal—*B. glandula* is already at the limit of its O_2_ delivery system during emersion stress or is effectively “maxed-out” in terms of its capacity for additional aerobic metabolic plasticity, and this could explain why we fail to see differences in parameters related to aerobic metabolism.

Another possible explanation for our CS and MO_2_ results can be found in the work of Castro et al. (2001), in which they revealed that the intertidal barnacle *J.cirratus* maintained relatively similar rates of O_2_ consumption regardless of their length of time in emersion. If air exposure duration does not affect the rate of O_2_ consumption in a closely-related, intertidal-adapted barnacle, this could explain why the MO_2_ of *B. glandula* does not differ between shore heights. Seasonal differences in food availability could also interfere with metabolic rates. In the California Current System, phytoplankton concentrations are typically high during the summer months—the main upwelling season—and low during the winter months (Thomas et al. 2009). Across taxa, food deprivation can trigger a reduction in baseline metabolic rates (Guppy and Withers 1999). During winter months when phytoplankton concentrations are low, an induced metabolic depression could have the effect of minimizing any possible tidal position differences in MO_2_. Barnacles from both the aquatic and aerial MO_2_ experiments, however, were collected during the summer and early fall months when chlorophyll levels in the seawater were high due to abundant phytoplankton (CENCOOS data portal; https://data.cencoos.org/). It is also conceivable that differences in aerobic capacity between barnacles from different tidal positions are relatively negligible under routine, non-stressful environmental conditions—as we observed—and it is only under stressful conditions (e.g., elevated temperatures) that differences become apparent. This hypothesis warrants further investigation.

While there were no effects of tidal position on MO_2_ in air or water, we did find that O_2_ consumption rates were approximately 3–4 times greater in humid air than in the water (Fig. 6). Higher O_2_ consumption rates in air relative to seawater have been observed in many intertidal organisms, including snails (Micallef and Bannister 1967), crabs (Wheatly and Taylor 1979), and other barnacle species (Petersen et al. 1974; Ober et al. 2019). Ober et al. (2019) reported that *B. glandula* had MO_2_ values up to 2 times greater in air than seawater, and the gooseneck barnacle *Pollicipes polymerus* has O_2_ consumption rates in air that were up to 5 times greater than in water (Petersen et al. 1974). Even some subtidal barnacles, such as *A. psittacus*, have high capacity for O_2_ consumption in air despite the fact that they rarely experience emersion conditions (López et al. 2003). Again, these data reveal the tremendous capacity for aerial O_2_ uptake possessed by acorn barnacles.

### Behavior

Barnacle behavior was the final process we measured in an effort to comprehensively characterize the metabolic phenotype of *B. glandula* across the intertidal zone. A description of the various forms of barnacle cirral activity has been thoroughly detailed by Crisp and Southward (1961). In this study, we quantified the percentage of time immersed barnacles from each tidal height spent with their operculum closed or their operculum open while engaged in each of the following behaviors: normal and fast cirral beating (full cirral extensions and retractions aimed at feeding), pumping (partial cirral extension and retraction with incomplete unfurling; typically considered a respiratory behavior), and testing (valves slightly open with no cirral extensions and very minor inward seawater flow; typically considered to be a behavior aimed at testing the conditions of the water). Of these behaviors, only cirral beating facilitates substantial food capture, though beating and pumping behaviors similarly increase water flow through the mantle cavity (Crisp and Southward 1961) and so are both considered to be respiratory in nature. We, therefore, considered these behaviors to generally reflect the metabolic demand of an individual barnacle.

We found that the barnacles from the upper intertidal positions were active (i.e., open and engaged in beating, pumping, or testing) significantly more often than those from the low intertidal positions, and that this increase in overall activity is largely the result of increases in the amount of time spent attempting to feed with a normal beat (Fig. 7A) and decreases in the amount of time spent testing. Since barnacles cannot feed during air emersion, it follows that those from the higher intertidal would spend an increased amount of time feeding whenever they are submerged, compared to lower intertidal barnacles that spend more time underwater. This trend has been shown for several intertidal species (e.g., barnacles (Ritz and Crisp 1970), bivalves (Ballantine and Morton 1956), and gastropods (Newell et al. 1971)), whereby organisms that are held in the air for increasing periods of time will increase the amount of time they spend feeding when subsequently re-immersed. Not only do higher intertidal barnacles feed more, but also there is evidence of morphological differences aimed at increasing food capture between conspecifics located at different tidal heights. Chan and Hung (2005) revealed that *Tetraclita japonica*, another barnacle with a wide vertical distribution, had longer cirri in barnacles in the upper portion of their vertical distribution, which would serve to enhance feeding ability during shorter durations of immersion. We did not find an effect of intertidal position on the amount of time spent pumping while submerged. This likely reflects the fact that previous air emersion is unlikely to cause a reduction in hemolymph pO_2_ for *B. glandula* at any tidal height and so increased efforts to reoxygenate through this respiratory behavior would be unnecessary regardless of differences in the duration of low-tide air emersion between each tidal position.

In addition to quantifying the duration of time spent performing each behavior, we also quantified beat frequencies for normal cirral beating and pumping behaviors in *B. glandula* from across the intertidal zone. We know that cirral beat frequency in barnacles is sensitive to both seawater flow rates and temperature, with maximum beat frequencies occurring at some optimal, species-specific flow rate and temperature (Southward 1955; Southward and Crisp 1965; Nishizaki and Carrington 2014a, 2014b). In our experiment, barnacle behavior was quantified within the respirometry chambers under conditions of relatively low velocity flow (∼13 cm^3^/s) and constant temperature (14°C), both of which fall within the optimal ranges for *B. glandula* cirral beating and food capture (Nishizaki and Carrington 2014a). We found that higher intertidal barnacles performed pumping beats significantly more slowly than individuals in the low intertidal, and though the trend was similar for normal beat frequency, the differences were not significant (Fig. 7). This is consistent with research by Southward (1955) who observed that the barnacles *Eliminus modestus, Chthamalus stellatus*, and *B.balanoides* all exhibited decreased cirral beat frequencies at higher shore heights compared to conspecifics at lower shore heights. (These differences between heights also disappeared after being held in the same laboratory conditions over 24 h, which supports our decision to observe barnacle behaviors immediately after field collection.) We argue that upper intertidal barnacles are sufficiently oxygenated by normal beating that occurs during increased bouts of feeding while in water, such that pumping activity, which predominantly serves a respiratory function, is not required at as high of a beat rate. Further, Gilman et al. (2013) found that respiration rates for *B. glandula* were not different between pumping and normal beats, so there would be no energetic cost-savings associated with using pumping rather than normal beating to acquire O_2_.

## Conclusion

As global climate change progresses, the intertidal zone is expected to experience even greater extremes in abiotic conditions like temperature, O_2_ availability, and wave action (Harley et al. 2006; Helmuth et al. 2006). To make predictions about the effects of climate change on the ecology of a system requires an understanding of the physiological effects of environmental stress on resident organisms, as we attempt to provide herein. Several frameworks have been put forth that present unifying physiological explanations to bridge climate stress with its ecosystem-level effects. The contentious OCLTT concept described above is one such example (Pörtner et al. 2017; Pörtner 2021). Comprehensive bioenergetics frameworks have also been detailed (Somero 2002; Sokolova et al. 2012; Sokolova 2013; Matzelle et al. 2015; Sokolova 2021), which assert that energy availability and energetic reallocation during environmental stress (from growth, reproduction, and/or development to basal maintenance) dictate tolerance limits broadly, particularly during multi-stressor exposure. And finally, relative physiological acclimation ability, when considered itself as a plastic trait, must be high enough in an organism for it to successfully acclimate to environmental stress events (Calosi et al. 2008; Gunderson and Stillman 2015; Seebacher et al. 2015). Intertidal organisms, especially those in the highest reaches of the intertidal where prolonged high temperatures can occur, may not have this capacity (Stillman 2003; Dillon et al. 2010; Somero 2010).

It is clear from these physiological frameworks that indices of metabolism, as well as their relative degree of plasticity, are important to consider when predicting how an organism will respond to environmental variation (e.g., Dillon et al. 2010), whether that variation is driven by climate change or relative intertidal position. Herein, we provide evidence for differences in some aspects of the metabolic phenotype of *B.glandula* barnacles residing in different intertidal zones. Specifically, we have shown that intertidal position primarily influences anaerobic capacity and cirral behavior, with barnacles from the high intertidal exhibiting a decreased capacity for anaerobic metabolism (with no overall differences in aerobic indicators), increased feeding behaviors during immersion, and a larger average body size compared to conspecifics in the low tidal zone. The relatively larger body size of high intertidal barnacles is counter to the bioenergetic models, which would predict that extreme stress in the high intertidal would come at the cost of growth and might result in smaller animals. Likewise, we see evidence for plasticity in parameters relating to anaerobic metabolism which suggests that *B. glandula* has not exceeded its capacity for stress acclimation. Together these data loosely suggest that *B. glandula* are not immediately threatened by climate stress.

To date, studies examining the effects of tidal position on metabolic parameters within a single species are virtually absent in the current literature (see Dowd et al. 2013), though works that compare metabolic indices between intertidal species can be found (e.g., Augenfeld 1967; Stillman and Somero 1996; Tomanek and Somero 1999; Vial et al. 1999; Gunderson et al. 2019). Our findings, therefore, represent a valuable contribution to the field of intertidal ecophysiology, climate change research, and conservation physiology. And the fact that we observed differences in the metabolic capacity of a single barnacle species over such relatively small spatial scales, highlights how variable the susceptibility to environmental stress can be within a single population. Metabolic data such as those we present herein could potentially be incorporated into predictive mathematical models being used to inform conservation efforts (e.g., O’Connor et al. 2007). As we move forward with this research, we are interested in characterizing the relative capacity for phenotypic plasticity exhibited by conspecifics at different positions in the intertidal. Metabolic data such as these could also prove useful to conservation efforts, as it is vital to know which organisms have the physiological plasticity necessary to acclimate (and ultimately adapt) to a rapidly changing environment (Costa and Sinervo 2004; Wikelski and Cooke 2006). If evidence of plasticity is not found, we will explore the possibility that selection during settlement is responsible for differences in metabolic phenotype between tidal positions and seek to identify underlying genotype differences between these populations (sensu Schmidt and Rand 2001).
